# Electrochemical Polarization of Disparate Catalytic Sites Drives Thermochemical Rate Enhancement

**DOI:** 10.1021/acscatal.3c03364

**Published:** 2023-10-20

**Authors:** Isaac T. Daniel, Bohyeon Kim, Mark Douthwaite, Samuel Pattisson, Richard J. Lewis, Joseph Cline, David J. Morgan, Donald Bethell, Christopher J. Kiely, Steven McIntosh, Graham J. Hutchings

**Affiliations:** † Max Planck-Cardiff Centre on the Fundamentals of Heterogeneous Catalysis FUNCAT, Cardiff Catalysis Institute, School of Chemistry, Cardiff University, Translational Research Hub, Cardiff CF24 4HQ, U.K.; ‡ Department of Chemical and Biomolecular Engineering, Lehigh University, Bethlehem, Pennsylvania 18015, United States; § Department of Materials Science and Engineering, Lehigh University, Bethlehem, Pennsylvania 18015, United States

**Keywords:** bimetallic catalysis, redox, electron transfer, oxidative dehydrogenation, electrochemical polarization, rate enhancement

## Abstract

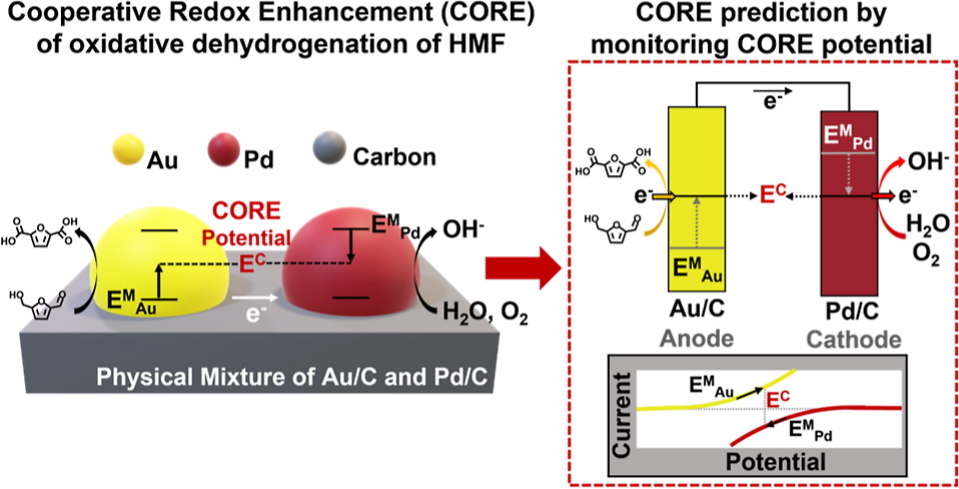

Supported bimetallic catalysts commonly exhibit higher rates of reaction compared to their monometallic counterparts, but the origin of these enhancements is often poorly defined. The recent discovery that cooperative redox enhancement effects in Au–Pd systems promote bimetallic catalysis in thermochemical oxidation is an important development in this field. This effect aligns two important research fields, thermo- and electrocatalysis, but questions relating to the generality and origin of the effect remain. Here, we demonstrate that these effects can be observed in reactions over a range of bimetal combinations and reveal the origin using a combination of electrochemical and material characterization. We disclose that the observed activity enhancement in thermochemical systems is a result of the electrochemical polarization of two disparate catalytic sites. This forms an alternative operating potential for a given bimetallic system that increases the driving force of each of the composite half reactions in oxidative dehydrogenation. We therefore uncover the physicochemical descriptors that dictate whether these enhancement effects will be exhibited by a particular combination of supported metal catalysts and determine the magnitude of the effect.

## Introduction

It has been extensively demonstrated that combining metals to produce supported multimetallic catalysts is an effective method of increasing the catalyst performance.^1−3^ Recently, we reported a new catalytic effect for reactions over nonalloyed bimetallic systems.^4^ We demonstrated that a physical mixture of supported Au and Pd catalysts was considerably more active toward alcohol and formyl oxidative dehydrogenation (ODH) compared to the sum of their independent monometallic activities or that of an equivalent alloy composition. This observation was attributed to cooperative redox enhancement (CORE) effects, which is substantially different from previous arguments used to explain synergistic interactions exhibited by supported bimetallic catalysts, such as electronic and geometric effects.

CORE is governed by the coupling of two individual half-cell reactions: the oxygen reduction reaction (ORR) and dehydrogenation (DH), which are catalyzed by spatially separated, but electrochemically connected, supported catalytic sites. The differential activity of each catalyst toward the ORR or DH was used to explain the rate enhancement observed upon the electrochemical coupling of the Au and Pd metal components.^4^ The separated metal particles independently contribute high activity for the half-reactions but, in the thermal system, are coupled via electron transport through a conductive support. We have since demonstrated that the magnitude of the CORE effect can be influenced by the atomic ratio of Au and Pd present^5^ and the size of the supported metal particles.^6^

The electrochemical coupling of two half-reactions in monometallic or alloyed catalysts has been experimentally demonstrated to occur in various thermochemical systems, including liquid phase oxidation.^7,8^ Recently, mixed potential theory, which will be discussed in more detail later in this work, has been used to demonstrate that the activity and selectivity of supported metal nanoparticles toward H_2_O_2_ production can be predicted by the electrochemical measurement of the composite individual half-reactions.^9^ Using a similar analysis, it has been shown how two coupled half-reactions in a single catalyst can describe the thermochemical activity of both the aerobic oxidation of formic acid and hydroquinone.^10,11^ This analysis is not just limited to oxidation, with the hydrogenation of 4-nitrophenol also being well described by an electron-transfer mechanism.^12^ These studies demonstrate how the electrochemical mechanism that underpins the CORE effects is applicable in a multitude of thermochemical scenarios. Despite the existing bank of literature that supports the underlying mechanism, the fundamental understanding of CORE which, importantly, happens when two dissimilar catalysts are present in one system, has not been documented previously. Herein, we propose a fundamental electrochemical mechanism to describe such effects and, furthermore, provide the foundation for a methodology to predict the magnitude of such CORE effects in thermo-catalytic systems for various supported metal catalysts.

## Results and Discussion

Initially, a series of monometallic Au, Pd, and Pt catalysts supported on Vulcan XC72-R carbon were synthesized using a sol-immobilization procedure,^13,14^ along with an analogous Ir catalyst synthesized by impregnation,^15^ to achieve the desired metal loading (1 wt %). The actual metal loading of each catalyst was confirmed by inductively coupled plasma mass spectrometry (ICP–MS) (Table S1). Pt catalysts are well studied for ODH and exhibit excellent performance toward the oxidation of 5-hydroxymethylfurfural (HMF),^16−18^ the substrate that is the focus of this work. Ir catalysts have also been effectively employed in the aerobic oxidation of several common substrates.^19−21^ The activity of Pt and Ir toward the ORR^22−24^ is also essential if CORE is to be realized for bimetal combinations utilizing these elements. Pt demonstrates high activity toward ORR, which is attributed to intermediate oxygen binding energies,^25−31^ and Ir is frequently used as a component in alloyed catalysts for the ORR.^31,32^

**Figure 1:**
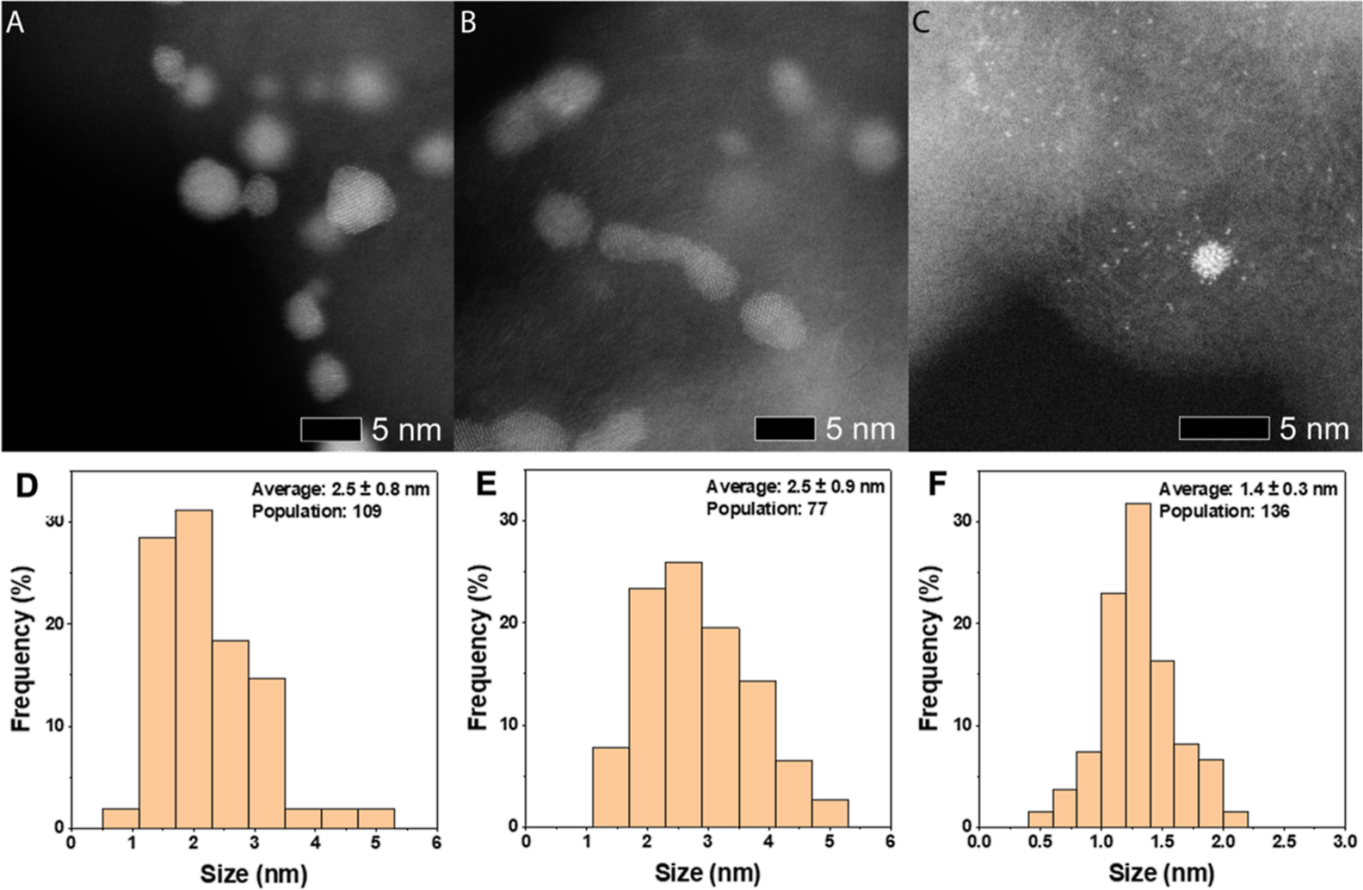
Representative HAADF STEM images of ca. 1 wt % monometallic catalysts (A) Au/C, (B) Pd/C, and (C) Pt/C as well as measured particle size distributions of (D) Au/C, (E) Pd/C, and (F) Pt/C (showing both nanoparticles and atomically dispersed Pt species). These catalysts were prepared by sol immobilization with average particle sizes and population counts displayed on the respective particle size distribution histograms.

**Figure 2:**
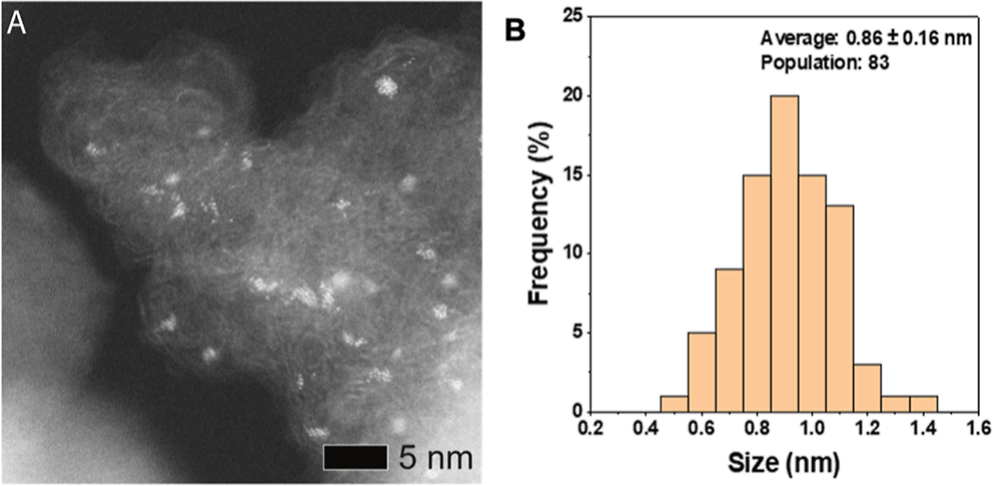
(A) Representative HAADF STEM image of ca. 1 wt % monometallic Ir/C. (B) Corresponding measured particle size distribution. The Ir/C catalyst was prepared by the impregnation methodology, with average particle sizes and population counts displayed in the particle size distribution histogram.

Each of the monometallic catalysts was analyzed by high angular dark field (HAADF) imaging in a scanning transmission electron microscope (STEM) and by X-ray photoelectron spectroscopy (XPS) to characterize their particle size distribution and electronic state (Figures 1, 2 and S1). Notably, Ir was the only supported metal that did not exist in a metallic state, although supported Ir-oxide has previously been demonstrated to be the active species in the aerobic oxidation of alcohols.^19^

For consistency with our previous studies on this topic,^4−6^ the ODH of HMF was selected as the model reaction. This involves three oxidations and terminates at 2,5-furandicarboxylic acid, an important platform chemical.^33−36^ The oxidation can proceed via two routes, meaning that there are three possible intermediates: namely, 2,5-diformylfuran (DFF), 5-hydroxymethyl-2-furancarboxylic acid (HMFCA), and 5-formyl-2-furancarboxylic acid (FFCA) (Scheme S1).

**Figure 3:**
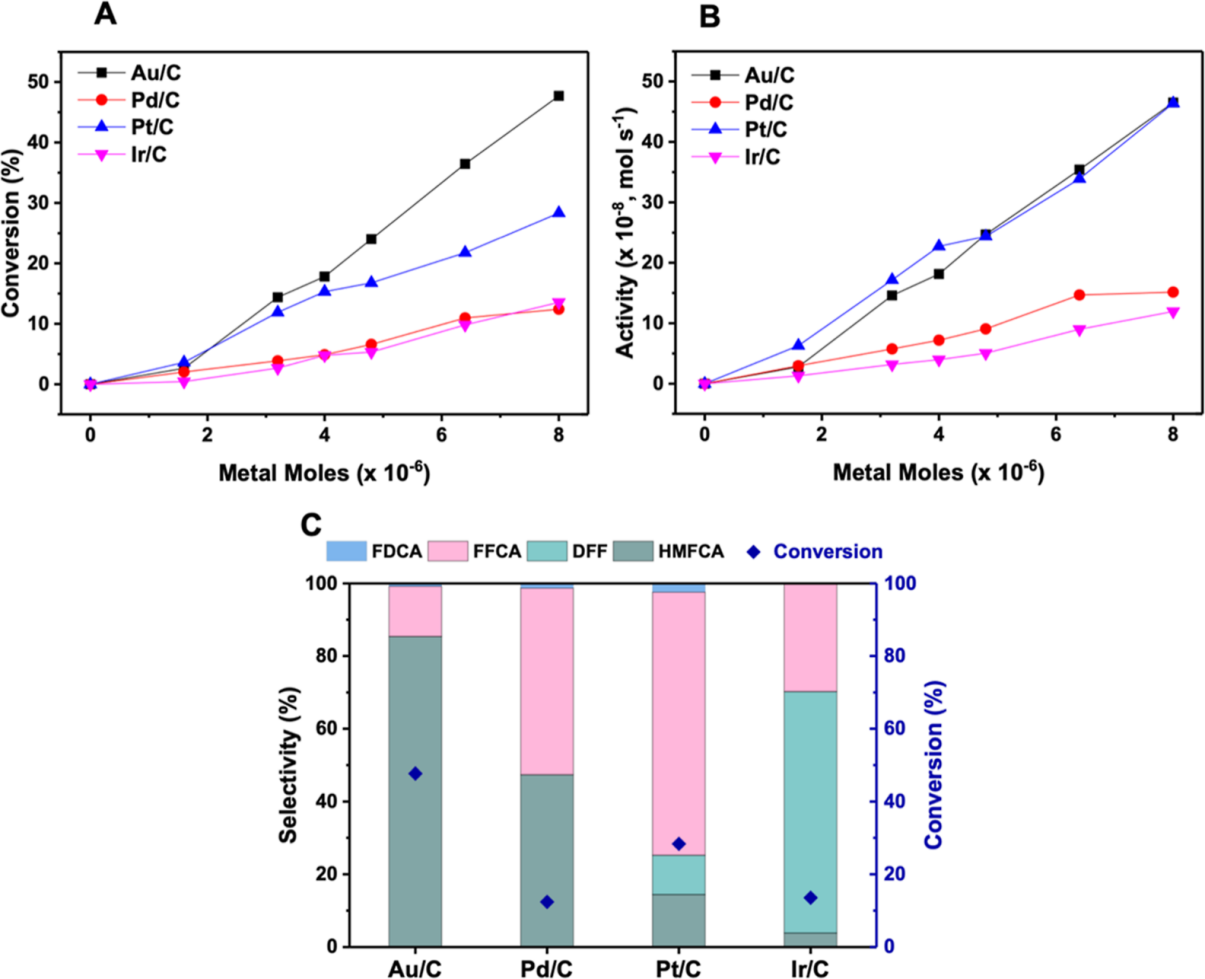
Thermo-catalytic performance of monometallic catalysts. (A) HMF conversion and (B) activity for the stated monometallic catalytic systems at various molar quantities. The number of moles of each metal present is varied by changing the mass of catalyst added to the reaction. (C) Selectivity compared to HMF conversion at 8 × 10^–6^ moles of each metal. Reaction conditions: H_2_O (16 mL), HMF (0.1 M), NaHCO_3_ (0.4 M), 3 bar O_2_, 80 °C, 30 min. Metal loading of all catalysts is approximately 1 wt %, with the amount of metal moles in the reaction altered by varying the mass of each catalyst.

To obtain activity benchmarks, thermo-catalytic HMF oxidation experiments were first conducted over varying amounts of the four monometallic catalysts (Figure 3A,B). The catalyst performance was assessed under batch-slurry conditions using glass Colaver reactors (Scheme S2). The substrate/metal ratio was varied by changing the mass of each catalyst used. The strong correlation between catalyst mass and activity indicates that there is no evidence of solid/liquid mass transfer. The linear relationship is maintained even at high metal content, demonstrating that even at these quantities the occurring over the catalyst surface can still be examined. Reaction selectivity (Figure 3C) indicates that the Au/C catalyst strongly favors aldehyde oxidation, with HMFCA accounting for 85% of the products after 30 min of reaction. Meanwhile, the secondary transformation to FFCA is much more favorable over the Pt/C catalyst (72% of products), showing a preference for alcohol DH that is consistent with the literature^37,38^ and explains its higher turnover activity at higher catalyst quantities. Notably, different rates of HMF conversion and chemoselectivity were exhibited by each of the monometallic catalysts.

We previously proposed that CORE occurs via electrochemical redox coupling between two spatially separated but electrochemically connected particles, with each performing one-half-reaction.^4^ Borrowing from corrosion science, the conservation of charge is maintained when the system rests at the mixed potential (*E*^M^). This occurs where the rates of the anodic half-reaction, HMF oxidation (HMFOR, equivalent to thermo-catalytic DH), and cathodic ORR are balanced.^39^*E*^M^ is dictated by the onset potentials and overpotentials of the electrocatalytic metal, such that the corresponding mixed current density (*j*^M^) is a direct measure of activity.

**Figure 4:**
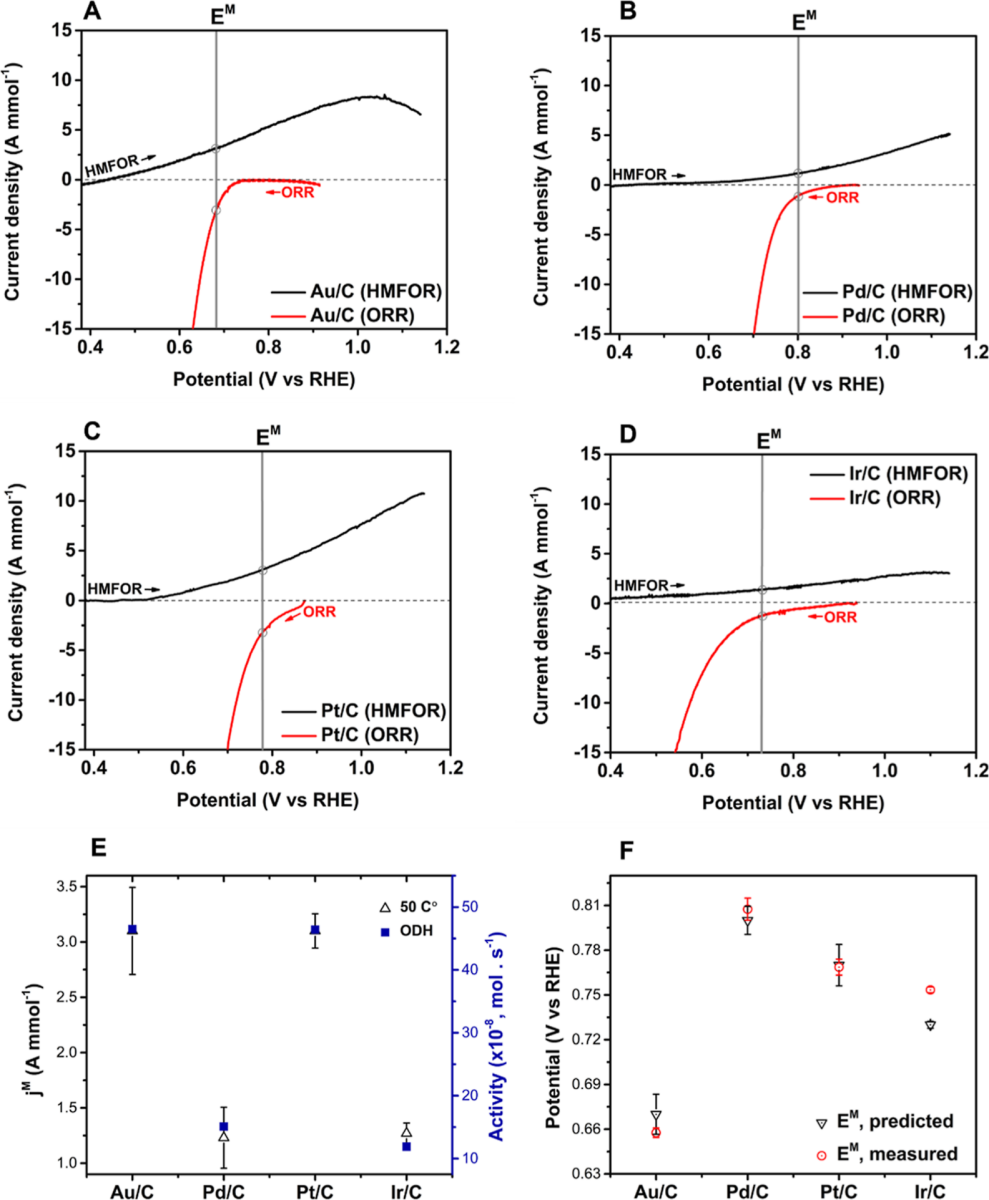
Electrocatalytic performance of monometallic catalysts for each half-reaction. LSV curves of monometallic catalytic systems (A) Au/C, (B) Pd/C, (C) Pt/C, and (D) Ir/C for HMFOR (black line) and ORR (red line). Reaction conditions (HMFOR): NaHCO_3_ (0.4 M), HMF (0.1 M), and N_2_ (50 mL min^–1^). Reaction conditions (ORR): NaHCO_3_ (0.4 M) and O_2_ (50 mL min^–1^). (E) Comparison between the *j*^M^ and thermo-catalytic ODH activity at 50 °C. (F) Comparison of predicted *E*^M^ and measured *E*^M^ at 50 °C. Reaction conditions (measured *E*^M^): NaHCO_3_ (0.4 M), HMF (0.1 M), and 50 mL min^–1^ of O_2_. All experiments were conducted at 50 °C. The error bars are calculated based on three or more independent experimental measurements.

Linear sweep voltammetry (LSV) curves were measured for each catalyst at 50 °C for HMFOR in the absence of O_2_ and the ORR in the absence of HMF (Figure 4). Taking monometallic Au/C as an example, HMFOR and ORR onset potentials are 0.47 and 0.72 V, respectively, with an *E*^M^ of 0.67 V and a corresponding *j*^M^ of 3.1 A mmol^–1^ (vertical line in Figure 4A). Identical measurements were performed for the Pd/C, Pt/C, and Ir/C catalysts. The onset potentials of the Pd/C catalyst were found to be 0.84 and 0.68 V for ORR and HMFOR, respectively, in good agreement with our previous work.^4^ Note that the Pt/C catalyst provides the highest ORR and HMFOR activities among the catalysts investigated. All of the catalysts have regions of potential overlap between HMFOR and ORR, enabling the calculation of *E*^M^ and *j*^M^ (Table S2). The trends of HMFOR and ORR activity of the catalysts were maintained at room temperature (Figure S2).

Strikingly, the predicted values of *j*^M^ for each of the catalysts are extremely well-aligned with the thermo-catalytic ODH activity (Figures 4E and S2E). This means that the activity of the thermo-catalytic system, whereby separated metal nanoparticles are connected by a conductive support, is well captured by considering the reaction to occur as two coupled electrochemical half-reactions and by characterizing these half-reactions in an electrochemical cell. While precisely matching the reaction conditions employed for thermo-catalytic ODH (80 °C and 3 bar O_2_) is very challenging in an electrochemical system due to the stability of common reference electrodes,^40^ the strong correlation presented here indicates that our ambient pressure and lower temperature analyses effectively describe the thermo-catalytic system. In addition, the estimated thermo-catalytic activity (12.9 × 10^–8^ mol s^–1^ for Au/C) calculated from j^M^ shows the same order of magnitude compared to the measured activity (46.5 × 10^–8^ mol s^–1^), strongly supporting the notion that the electrochemical descriptors represent the monometallic thermo-catalytic system despite the inevitable differences in conditions. Ultimately, these experiments indicate that *E*^M^ and *j*^M^ are applicable descriptors for higher temperature oxidations of larger organic molecules, which to our knowledge has only previously been shown for less complex substrates.^9,10,39,41,42^

An unpolarized electrocatalyst particle exposed to both HMF and O_2_ is under essentially the same conditions as a thermo-catalyst particle. If a bimetallic system is simultaneously active for both half-reactions, we would expect the resting potential of the electrocatalyst particle to be the *E*^M^ shown in Figure 4 because HMFOR and ORR occur simultaneously, and at balanced rates, at the unpolarized electrode potential. Measuring the *E*^M^ of the electrodes, in the presence of both HMF and O_2_, provides near-perfect agreement (within experimental error) between the measured and predicted *E*^M^ values (Figure 4F and Table S2). Furthermore, employing Tafel analysis, a common alternative approach to measure *E*^M^ and *j*^M^ in the presence of coreactants,^43^ again shows excellent alignment with the thermo-catalytic data (Figure S3). The alignment of the thermo-catalytic activities with electrocatalytic data strongly supports the hypothesis that the overall HMF oxidation reaction occurs via electrochemically coupled half-reactions. In turn, this supports the hypothesis that this coupling is the origin of CORE and indicates crucially that this electrochemical approach can be extended to predict the presence or absence of the CORE effect in multimetallic thermo-catalytic systems.

**Figure 5:**
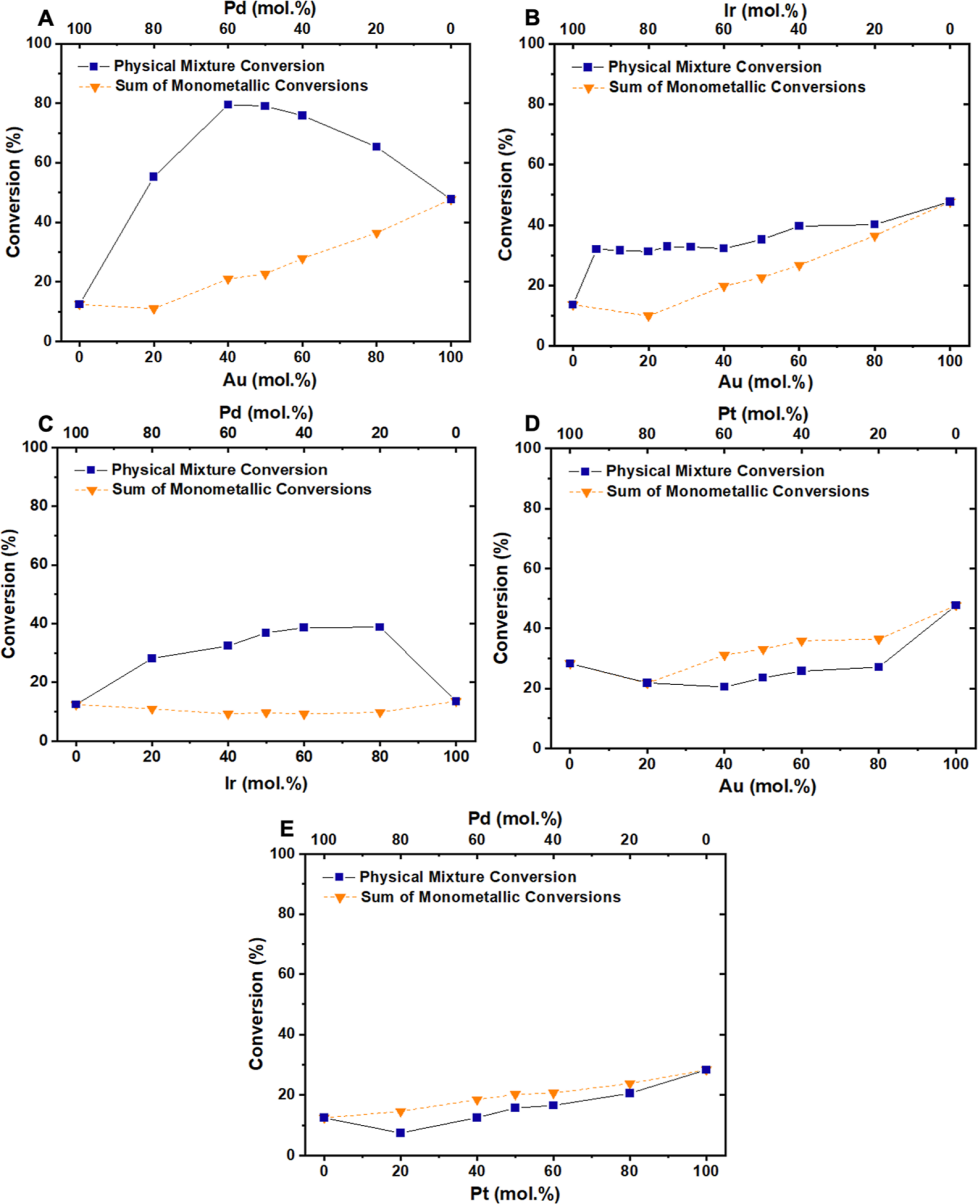
Thermo-catalytic physical mixture systems consisting of two monometallic catalysts showing HMF conversion as compared to the sum of their monometallic analogues. (A) Au/C + Pd/C, reproduced with permission,^5^ (B) Au/C + Ir/C, (C) Pd/C + Ir/C, (D) Au/C + Pt/C, and (E) Pt/C + Pd/C. Reaction conditions: H_2_O (16 mL), HMF (0.1 M), NaHCO_3_ (0.4 M), 3 bar O_2_, 80 °C, 30 min. All catalysts are approximately 1 wt %, and in the bimetallic systems, the total metal content is constant [HMF: metal = 200:1 (mol: mol)]. The sum of the monometallic conversions is taken from the monometallic data of each respective metal, for the specific molar quantity.

Thermo-catalytic HMF oxidation was assessed in reactions over physical mixtures of pairs of monometallic catalysts (Figure 5). In each case, the influence of the component metal ratio was also probed; a parameter that, with some metal combinations, had a profound effect on the magnitude of the CORE observed. The conversion exhibited by each physical mixture is compared to the sum of the corresponding monometallic systems, at each respective metal quantity.

The influence of the Au: Pd ratio on the magnitude of CORE has already been reported^5^ but is reproduced here to aid comparison with the other physical mixtures under investigation. In binary physical mixtures of Au/C, Pd/C, and Ir/C (Figure 5B,C), increased HMF conversion is exhibited compared to the sum of the monometallic systems, thus confirming that the CORE effect is also present in these systems and that the effect is not specific to Au and Pd catalysts. Previous analysis of these systems has shown that leaching and migration of Au and Pd does not occur under these reaction conditions.^4^ Activity comparisons for the Ir-containing physical mixture systems similarly show a CORE enhancement, and as with the monometallic systems (Figure 3C), there are clear differences in selectivity and reaction pathway between the physical mixture systems (Figure S4). Interestingly, physical mixtures containing Pt/C (Figure 5D,E) exhibited a decreased HMF conversion relative to the sum of the monometallic systems across all metal ratios. These results at first sight are somewhat surprising, given that supported Pt catalysts are known to be extremely active for both half-reactions.^13,24^

Following the demonstration that the mixed potential analysis, based on well-established corrosion science,^39,44,45^ accurately characterizes the monometallic systems, we have applied the same approach to the bimetallic physical mixtures, to explain the relative thermo-catalytic activities of the various systems. This analysis predicts that when two electrocatalysts with different *E*^M^ values are connected through a conductive support, such as Vulcan XC72-R, a new operating mixed potential (*E*^CORE^), and corresponding mixed current density (*j*^CORE^), are realized. This potential lies above the *E*^M^ of the lower potential, anodic catalyst material, and below the *E*^M^ of the higher potential, cathodic catalyst material. If realized, this would increase the operating potential for the anodic catalyst and drive the HMFOR at an increased rate, and similarly, decrease the operating potential of the cathodic catalyst to drive the ORR at an increased rate. We propose that this is the origin of the CORE effect—the electrochemical coupling between the catalysts leads to increased driving forces for the half-reactions and thus an enhanced reaction rate.

**Figure 6:**
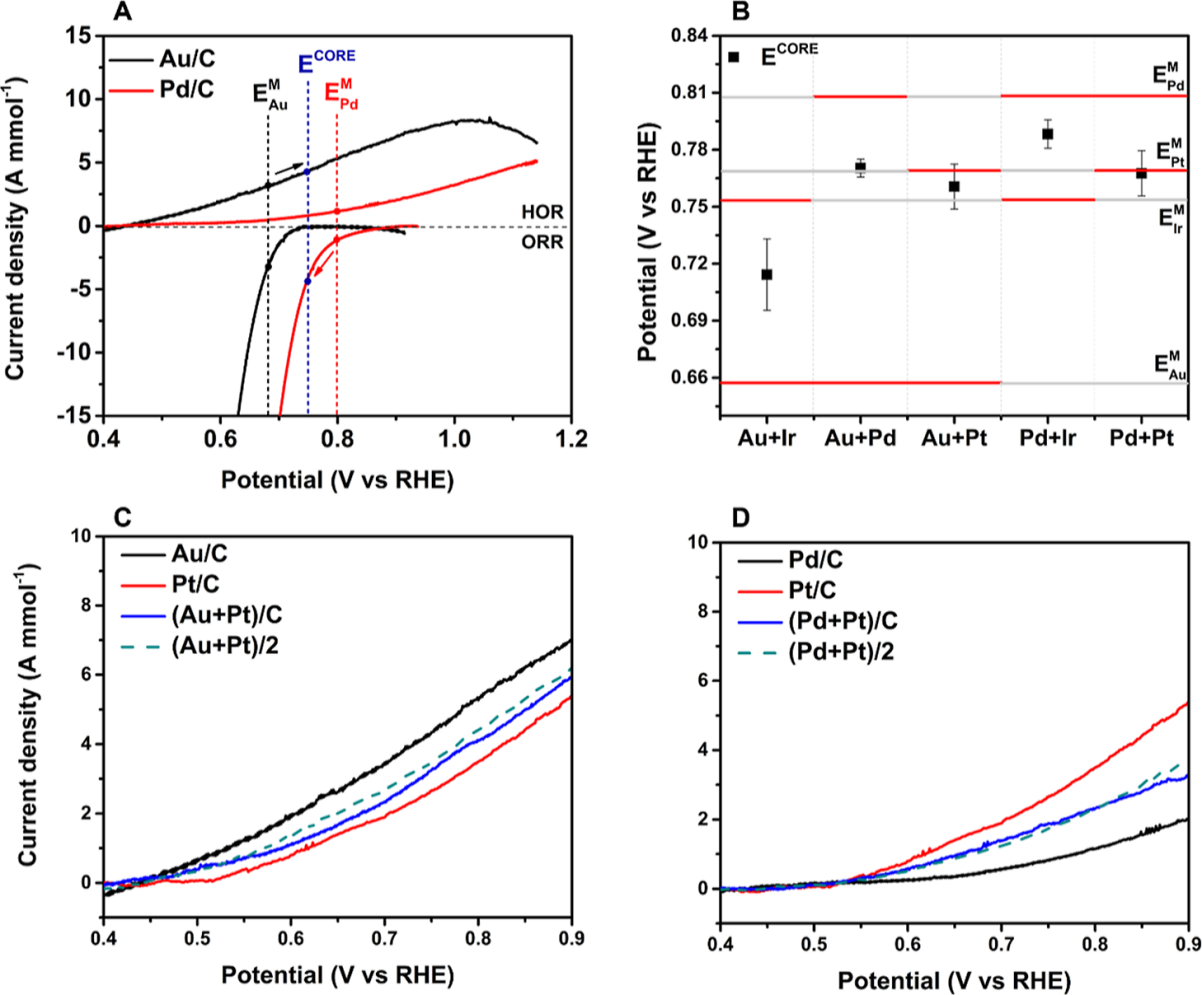
Electrocatalytic bimetallic systems (A) HMFOR and ORR LSVs of Au/C (black line) and Pd/C (red line). Reaction conditions (HMFOR): NaHCO_3_, 0.1 M HMF, 50 mL min^–1^ of N_2_, and 50 °C. Reaction conditions (ORR): NaHCO_3_ (0.4 M), O_2_ (50 mL min^–1^), 50 °C. (B) Comparison of measured *E*^M^ and *E*^CORE^ of monometallic and bimetallic mixture catalysts under identical reaction conditions. HMFOR LSVs of (C) Au/C, Pt/C, and Au/C + Pt/C; (D) Pd/C, Pt/C, and Pt/C + Pd/C. The average value for the stated monometallic catalysts is shown by dashed lines. Reaction conditions: 0.4 M NaHCO_3_ (0.4 M), HMF (0.1 M), N_2_ (50 mL min^–1^), 50 °C. The error bars are calculated based on three or more independent experimental measurements.

Electrocatalytic measurements of these physical mixture components support this hypothesized origin of the CORE effect. Note that a fixed metal ratio of 1:1 (mol: mol) is used in all electrochemical studies. Utilizing the Au and Pd system as an example, we would predict that, when coupled, rate enhanced HMF oxidation and ORR will occur on Au and Pd, respectively (Figure 6A). The CORE potential, *E*^CORE^, lies above the *E*^M^ for Au, thus positively polarizing the Au (Figure 6A, black arrow) and driving the rate of HMFOR on Au. Similarly, *E*^CORE^ lies below the *E*^M^ for Pd, thus negatively polarizing the Pd (Figure 6A, red arrow) and driving the rate of the ORR on Pd. The corresponding CORE current density, *j*^CORE^, is thus predicted to be higher than the individual *j*^M^ of either monometallic catalyst. As previously reported with extensive analysis, the overall rate of HMF oxidation is higher for a physical mixture of Au and Pd catalysts when compared to the monometallic systems.^4,5^ Thus, the trend predicted from this electrochemical analysis holds true in the CORE-enhanced thermo-catalytic behavior. However, it is worth noting that not all thermo-catalytic reaction turnovers would be operated by the electrochemical coupling between the catalysts, given realistic reaction conditions.

Extending to the other bimetallic metal systems, the three systems that exhibit the thermo-catalytic CORE effect (Au/C + Pd/C, Pd/C + Ir/C, and Au/C + Ir/C) all show *E*^CORE^ values located between the two respective *E*^M^ values (Figure 6B), indicating that they are all operating under this same electrochemical regime. Thus, our electrochemical explanation for the CORE holds true across differing materials, showing the generality of the effect.

To further highlight how the electrochemical analysis can describe the thermo-catalytic activities, we can consider the effect that changing the molar ratio of metals has on CORE. With the Ir-containing systems, the maximum CORE is observed when Ir is in excess, indicating that the half-reaction occurring over this catalyst must be rate limiting, which is supported by electrocatalytic measurements (Table S3 and Figure S5). In the Au/C + Ir/C system (Figure 5B), the absolute HMF conversion is relatively consistent (31–35%) between 5 and 50 Au mol %. Given that Au is responsible for catalyzing the DH half-reaction in this combination (indicated by comparison of the relative onset potentials, Table S2), as the Au content is increased from 5 to 50 mol %, the intrinsic HMF conversion must be limited by another factor. As such, the rate of the ORR occurring over the Ir/C catalyst must be limiting the overall activity of the system. This is consistent with previous observations in the Au/C + Pd/C CORE system, whereby the availability of Pd sites, and consequentially the rate of the ORR, can limit the overall HMF conversion.^4^ Considering that supported Ir catalysts are generally poorer at catalyzing the ORR compared to supported Pd catalysts,^29^ the lower HMF conversion is justified (Figure S5A). The coupling between Au/C and Ir/C is also evidenced by the shift in selectivity from the monometallic data (Figure 3C) as the Au catalyst predominately performs the DH half-reaction. The production of DFF is decreased as the Au content increases due to the formation of HMFCA being more favorable over Au sites (Figure S4).

Lower intrinsic HMF conversions in the Pd/C + Ir/C physical mixture relative to the Au/C + Pd/C physical mixture can be explained in a similar fashion by considering the lower activity of Ir toward HMF DH compared to that of Au (Figure S5). This demonstrates that CORE effects are general and consistent across differing metallic combinations.

**Figure 7:**
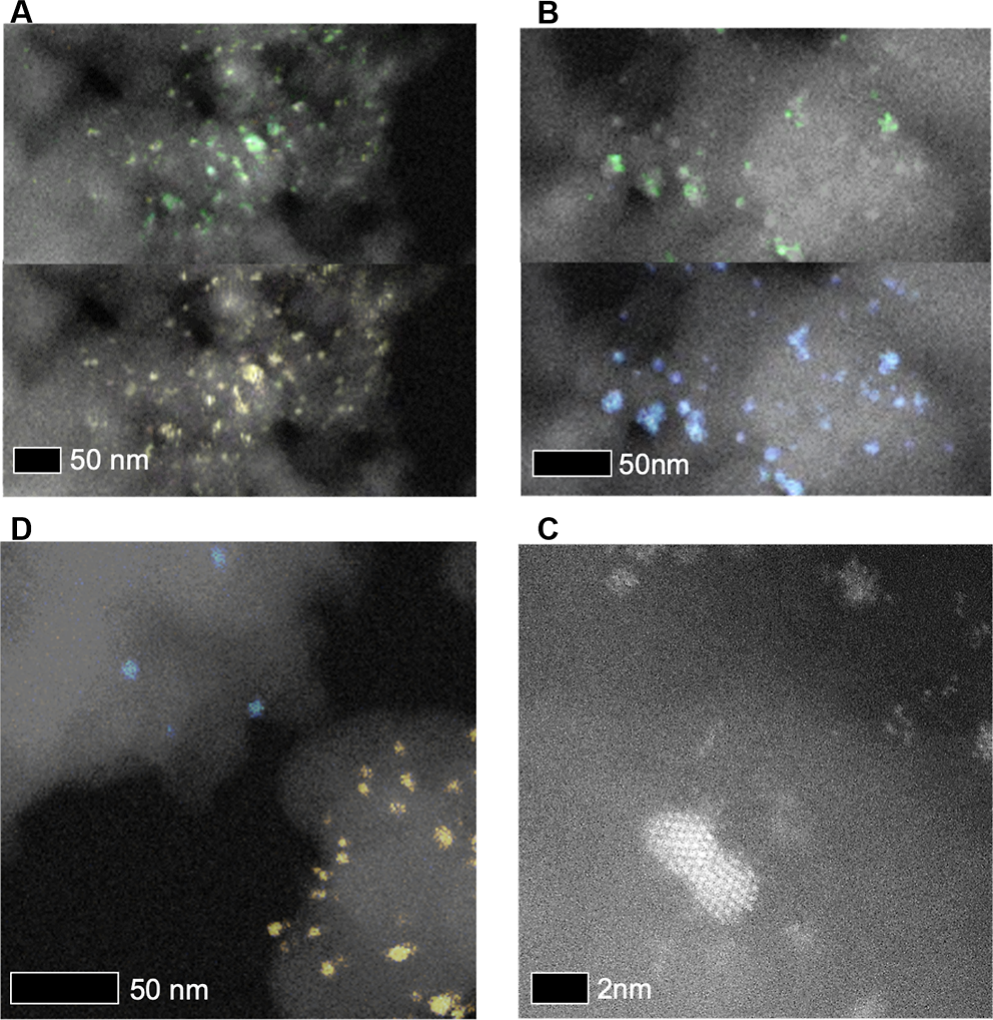
Representative HAADF STEM images with overlaid XEDS maps of postreaction catalysts involving a physical mixture of two monometallic catalysts. Color key: yellow = Au, green = Pt; and blue = Pd. (A) Au/C + Pt/C (upper panel, Pt map; lower panel, same area Au map) showing alloy formation; (B) Pd/C + Pt/C (upper panel, Pt map; lower panel, same area Pd map) showing alloy formation; (C) Au/C + Pd/C showing that physical separation of both monometallic components is maintained; (D) Au/C + Ir/C XEDS mapping not possible but sub nm Ir particles were still visible adjacent to, but separate from, the Au particles. All samples have an equimolar proportion of metal present and are approximately 1 wt %. Reaction conditions: H_2_O (16 mL), HMF (0.1 M), NaHCO_3_ (0.4 M), 3 bar O_2_, 80 °C, 30 min, HMF/metal = 200.

The Pt-containing systems that exhibit a negative effect when combined into a physical mixture (Figure 5D,E) have operating potentials almost identical to the measured *E*^M^ of pure Pt; 0.77 V (Pd/C + Pt/C) and 0.76 V (Au/C + Pt/C), respectively. Thus, when Pt is present, the rate of overall ODH is governed by the activity of Pt sites. This is further demonstrated by Pt-containing physical mixtures (Au/C + Pt/C; Pd/C + Pt/C) showing a current density that is very similar to an average of the respective monometallic systems, indicating that there is no electrochemical coupling and these mixtures do not directly affect the rate of HMF oxidation (Figure 6C,D). Furthermore, STEM analysis of postreaction catalysts indicates that in the Au/C + Pt/C and Pd/C + Pt/C systems, there is Pt migration, which results in the formation of alloyed particles (Figure 7A,B). XPS comparisons between pre- and postreaction Pt catalysts also show a shift to a lower binding energy, providing further evidence of alloying and Pt mobility (Figure S1).^46,47^ There is also an indication of partial Pt oxidation, which is common under reaction conditions such as these.

The mobility of Pt is key to explaining why a negative effect is seen in thermo-chemical systems. The formation of alloy nanoparticles effectively creates an entirely new catalytic species and removes the spatial separation between different metallic sites, which is vital to the CORE effect. It has previously been demonstrated that the formation of an alloy structure from a system containing two distinct phases reduces catalytic activity.^4^ Here, the new catalytic species is simply less active than the respective paired combination of monometallic catalysts. Where a positive CORE effect is observed in the original Au/C + Pd/C system, as well as the Ir-containing system, there is no evidence from postreaction analyses (STEM and XPS) of significant metal migration or alloying^4^ (Figures 7C,D, S1); hence, monometallic characteristics are preserved. This emphasizes the importance of maintaining spatial separation so that individual half-reactions can be coupled, causing polarization of each catalytic site and a subsequent increased reaction rate.

To summarize, the CORE potential, which can be derived from electrochemical analysis of catalyzed half reactions, can serve as a descriptor for the activity that is exhibited by multimetallic catalysts in thermochemical reactions. For this to be realized, the system must fit the following requirements: (1) the CORE potential must sit between the mixed potential of the two dissimilar metals involved; (2) the catalysts must exhibit different activities toward the oxidative and reductive half-reactions involved; and (3) there must be a region of overlap for the two component metals where both oxidation and reduction reactions can occur, without the presence of side reactions; and (4) the catalysts must be stable under reaction conditions so that the disparate sites are maintained and no metal transfer is observed.

## Conclusions

The CORE effect exhibited by various bimetallic systems arises from the electrochemical coupling of physically separated but electrochemically connected metal sites. The resulting shift in the operating potential experienced by each catalytic particle increases the driving force for each of the half-reactions, yielding higher rates. The thermal ODH of HMF over monometallic and bimetallic physical mixtures is well described by considering the electrochemical coupling of two half-reactions, as demonstrated by the excellent alignment of the electrochemical measurements with thermal ODH activities. The electrochemically derived CORE potential, *E*^CORE^, is an applicable and accurate predictor of the presence of the CORE effect in the thermo-chemical analysis. This relies upon *E*^CORE^ falling between the *E*^M^ of two dissimilar metals, where these metals have differential activity toward the component half-reactions and an area of potential overlap. The generality of the CORE effect and its origin is demonstrated by the fact that multiple metal combinations can be explained in the same fashion. Vital to observing these effects is the stability of the separated catalytic sites under reaction conditions, to ensure that coupling and subsequent polarization can occur. If these conditions are met, it is reasonable to state that electrochemical measurements can predict the activity of a general thermo-catalytic system that consists of two redox reactions.

## Supporting Information Available

Thermochemical, electrochemical, and catalyst analyses; XPS spectra for fresh and used catalyst samples, thermochemical activity, and selectivity data; electrochemical LSV curves for monometallic and bimetallic systems; and substrate conversion and experimental setup schemes (PDF)

## Abbreviations

ODH: oxidative dehydrogenation
ORR: oxygen reduction reaction
CORE: co-operative redox enhancement
DH: dehydrogenation
HMF: 5-hydroxymethylfurfural
HAADF: high angular dark field
STEM: scanning transmission electron microscopy
XPS: X-ray photoelectron spectroscopy
FDCA: 2,5-furandicarboxylic acid
DFF: diformylfuran
HMFCA: 5-hydroxymethyl-2-furancarboxylic acid
FFCA: 5-formyl-2-furancarboxylic acid
HMFOR: 5-hydroxymethyl furfural oxidation reaction
*E*^M^: mixed potential
*j*^M^: mixed current density
LSV: linear sweep voltammetry
