# Supplementary material for: Electrochemical Polarization of Disparate Catalytic Sites Drives Thermochemical Rate Enhancement
Source: ACS Catal. 2023 Oct 20;13(21):14189–98. doi: 10.1021/acscatal.3c03364 (PMC10631442; doi:10.1021/acscatal.3c03364)
Supplement: Supplementary file 1 — cs3c03364_si_001.pdf [file cs3c03364_si_001.pdf]

Supporting Information

Electrochemical polarization of disparate catalytic sites drives thermochemical rate enhancement.

*Isaac T. Daniel[1] †, Bohyeon Kim[2] †, Mark Douthwaite[1] *, Samuel Pattisson[1], Richard J. Lewis[1], Joseph Cline[3], David J. Morgan[1], Donald Bethell[1‡], Christopher J. Kiely[2,3], Steven McIntosh[2] *, and Graham J. Hutchings[1] **

[1]Max Planck-Cardiff Centre on the Fundamentals of Heterogeneous Catalysis FUNCAT, Cardiff Catalysis Institute, School of Chemistry, Cardiff University, Translational Research Hub, Cardiff, CF24 4HQ, UK.

[2]Department of Chemical and Biomolecular Engineering, Lehigh University, Bethlehem, PA, USA.

[3]Department of Materials Science and Engineering, Lehigh University, Bethlehem, PA, USA.

*Corresponding authors. Mark Douthwaite (douthwaitejm@cardiff.ac.uk), Steven McIntosh

(mcintosh@lehigh.edu) and Graham J. Hutchings (hutch@cardiff.ac.uk).

†Authors contributed equally.

‡Deceased 4th December 2022

Chemicals (Source, Purity) for thermocatalytic experiments

Chloroauric acid ($HAuCl_4 \cdot 3H_2O$, Strem Chemicals, 99.8%); distilled water (Millipore, 18.2 MΩ cm at 25 ˚C); 5-hydroxymethylfurfural (Sigma-Aldrich, >99%); palladium chloride ($PdCl_2$, Sigma-Aldrich, >99.9%); polyvinyl alcohol (PVA, Sigma-Aldrich, 80% hydrolyzed); sodium borohydride ($NaBH_4$, Sigma-Aldrich, 99.99%); sodium hydrogen carbonate (Fisher Scientific, >99.5%); Vulcan XC72R (Cabot); sulfuric acid (H_2SO_4, Fisher Scientific, ≥95%); chloroplatinic acid (H_2PtCl_6, Johnson Matthey, Assay 30.53%).

Chemicals (Source, Purity) for electrocatalytic experiments

Distilled water (18 MΩ cm), 5-formyl-2-furoic-acid (Tokyo Chemical Industry, 98%), 5-hydroxymethyl-2-furancarboxylic acid (Cayman Chemical, 98%), 5-hydroxymethylfurfural

(Sigma Aldrich, 99%), 2,5-furandicarboxylic acid (Sigma Aldrich, 97%), Nafion™ (Sigma Aldrich, 5 wt.% in alcohols and water), sodium bicarbonate (Fisher Scientific, 99.5%).

Catalyst synthesis

Two different synthesis methods were used to prepare the carbon supported, 1 wt.% catalysts in this study. Au, Pd and Pt catalysts were prepared by sol-immobilization; Ir catalysts were synthesized by impregnation as it was not possible to synthesize this catalyst using the same colloidal method. The theoretical weight loading for all the catalysts was 1 wt.% and the same batch of carbon support was used in all cases to ensure that the physical characteristics, such as high conductivity,[1,2] were constant across the sample set.

Sol immobilization

Known quantities of precursor, either $HAuCl_4$ (11.0 mg_{Au} mL^{-1}), $PdCl_2$ (5.5 mg_{Pd} mL^{-1}) or H_2PtCl_6 (15.3 mg_{Pt} mL^{-1}), and PVA (10 mg mL^{-1}, mass: metal mass = 1: 1) were added to vigorously stirred deionized water (350 mL). After 15 minutes, freshly prepared $NaBH_4$ solution (0.15 M, *mol : metal mol = 4 : 1 for Au and Pd, 8 : 1 for Pt*) was rapidly added and the resulting sol was stirred for 30 minutes. For Pt only, the sol was acidified by the addition of six drops of

concentrated H_2SO_4. The Vulcan XC72-R carbon support (1.98 g) was added followed by further mixing for 30 minutes to enable immobilization. The resulting catalyst was rinsed with excess deionized (DI) water (1 L g^{-1}), filtered and dried (110 ˚C, 16 h).

Impregnation

$IrCl_3$ (4.5 mg_{Ir} mL^{-1}) and DI water were added to a round bottom flask to a total volume of 16 mL. Under constant stirring, the temperature was raised to 65 ˚C and the Vulcan XC72-R carbon support (0.99 g) was added immediately. The temperature was further raised to 95 ˚C and the resulting slurry was left to stir for 2 h until a thick paste-like consistency was reached. The catalyst was dried (110 ˚C, 16 h) and reduced in a tube furnace at 300 ˚C for 4 h (ramp rate = 10 ˚C min^{-1}) under flowing 5% H_2/Ar (100 mL min^{-1}).

Catalyst characterization

Inductively Coupled Plasma Mass Spectrometry (ICP-MS) was used to determine metal loading. A known quantity of each catalyst sample was digested by microwave radiation in aqua regia (5 mL, 4 : 1 HNO_3 : HCl) prior to analysis. An Agilent 7900 ICP-MS with I-AS autosampler was used to analyze the digested catalysts for specific metal content. All samples were diluted by a

factor of 10 and made up with 500 μL of sample into class A volumetric flasks with DI-H_2O (which included 1% HNO_3 and 0.5% HCl matrix). For interference reduction, nickel sampling and skimmer cones were used with He mode on the ORS4 Octopol. Certified reference materials from Perkin Elmer (1000/100/10/10/blank μg L^{-1}) were used for five-point calibration, with an Agilent Certified Internal Standard.

Scanning Transmission Electron Microscopy (STEM) was conducted on an aberration corrected JEOL JEM-ARM200CF operating at 200 kV. Catalyst samples, dispersed on a holey carbon film supported on a 300 mesh copper grid, were imaged using a high angular annular dark field detector (HAADF). Energy Dispersive X-ray Spectroscopy (XEDS) was performed using a JEOL Centurion XEDS system for compositional analysis. Approximately 100 particles were measured for each catalyst sample to produce a statistically relevant particle size distribution.

X-ray photoelectron spectroscopy (XPS) was performed on a Thermo Fisher Scientific K-alpha+ spectrometer. Samples were analyzed using a micro-focused monochromatic Al X-ray source (72 W) using the "400-mm spot" mode, which provides an analysis defining elliptical X-ray spot of *ca.* 400 x 600 mm. Data was recorded at pass energies of 150 eV for survey scans and 40 eV for high resolution scan with 1 eV and 0.1 eV step sizes respectively. Charge neutralization of the

sample was achieved using a combination of both low energy electrons and argon ions. Data analysis was performed in CasaXPS v2.3.25 after calibrating the data to the C(1*s*) peak maximum at 284.5 eV, which is characteristic of the sp^2 component of the activated carbon.[3] Quantification was made using a Shirley type background and Scofield cross-sections, with an electron energy dependence corresponding to the TPP-2M method.[4]

Thermocatalytic testing and analysis

The oxidative dehydrogenation of 5-hydroxymethylfurfural (HMF) was used as a model reaction throughout to analyze catalyst activity and a schematic of the setup is provided in Scheme S2. HMF (0.2 M, 8 mL) and $NaHCO_3$ (0.8 M, 8 mL) were charged to a 50 mL glass Colaver reactor and stirred (1000 rpm) for 10 minutes at 80 ˚C. Following temperature stabilization, the required quantity of catalyst was added, and the reactor was purged 5 times with O_2, after which pressure was maintained at 3 bar. The typical reaction time was 30 minutes with samples taken at regular intervals. Analysis of post-reaction solutions was carried out using High-Performance Liquid Chromatography (Agilent Technologies 1200 series) following a thirty-fold dilution. A diode array detector and Hi-Plex H (300 x 7.7 mm) column were utilized with a mobile phase (0.7 mL min^{-1}) of dilute H_2SO_4 (5.5 mM). Pre-determined response factors based on calibrations of known quantities of HMF, 5-hydroxymethyl-2-furancarboxylic acid (HMFCA), 2,5-diformylfuran (DFF),

5-formyl-2-furancarboxylic acid (FFCA) and 2,5-furandicarboxylic acid (FDCA) were used to accurately quantify conversion (Eq. 1), selectivity (Eq. 2), and turnover activity (Eq. 3).

$$\text{HMF Conversion (time t, \%)} = \frac{\text{moles}_{\text{initial}} - \text{moles}_{\text{t}}}{\text{moles}_{\text{initial}}} \times 100 \#(1)$$

$$\text{Selectivity (product x, \%)} = \frac{\text{moles}_{\text{x}}}{\Sigma\ \text{moles}_{\text{x,y,z}}} \times 100 \#(2)$$

$$\text{Activity (mol s}^{-1}) = \frac{(\text{moles}_{\text{HMFCA}} \times 1) + (\text{moles}_{DFF} \times 1) + (\text{moles}_{\text{FFCA}} \times 2) + (\text{moles}_{\text{FDCA}} \times 3)}{\text{Time (s)}} \#(3)$$

Electrocatalytic testing

Error bars on electrochemical results are determined from three or more independent data sets. As the ODH activities were normalized by mmol of metals, the current generated from electrochemical reactions was also normalized by mmol of metal.

Preparation of electrodes

Before catalyst coating, indium tin oxide (ITO) coated glasses (8-12 Ω sq^{-1}) were washed with ethanol and DI water several times, before being sonicated with DI water to remove surface contaminants. A catalyst ink was prepared by ultrasonication for 150 seconds with a mixture of a monometallic catalyst (7 mg), DI water (1 mL), and Nafion solution (0.1 mL). A few mL of the

catalyst ink (containing 6.46 × 10^{-6} mmol of metal) were dropped onto the ITO glass (1 cm × 5 cm) and dried for 20 h at room temperature. When using a bimetallic, physical mixture system, two different catalyst inks containing the same amount of requisite metal (1 : 1 molar ratio) were prepared and mixed by ultrasonication for 60 s. The droplet of the mixed catalyst ink contained the same total mmol amount of metals as in the monometallic case.

Catalytic testing conditions

All electrochemical experiments were performed using a standard three-electrode electrochemical cell (50 mL) with sodium bicarbonate (0.4 M) electrolyte, with or without addition of HMF (0.1 M). All experiments were conducted under an oxygen or nitrogen environment with a flow rate of 50 mL s^{-1}. A Hg/HgO (in 1 M NaOH) reference electrode was utilized for the room temperature measurements and a homemade Ag/AgCl (in 1 M KCl) reference electrode was utilized for the elevated temperature measurements. A Pt wire was used as the counter electrode. All measured electrochemical potential values were converted with respect to the reversible hydrogen electrode (RHE) using Equation 4. A schematic of the electrochemical setups used is given in Scheme S3.

$$E_{RHE} = E_{Ref} + 0.059\ (pH) + E^{O}_{Ref}\ \#\ (4)$$

Activation of coated catalysts

Activation of catalysts was conducted when the catalyst-coated ITO was first submerged into a solution. This process removes adsorbed gases and any impurities attached to the catalytic surface. The electrolyte with $NaHCO_3$ was purged with O_2 and prepared electrodes were activated by cyclic voltammetry (0.8 V to 0.3 V vs RHE) under oxygen. The solution was then re-purged for 30 mins with N_2 or O_2, depending on the measurement.

Measured E^C and E^M, and Tafel analysis

The measured E^C and E^M were recorded using the same experimental apparatus for the monometallic and physical mixture catalytic systems (Supplementary Scheme 2). A solution containing HMF (0.1 M) and $NaHCO_3$ (0.4 M) was purged with oxygen and an activated catalyst was submerged into the solution. An open circuit potential mode was applied to monitor the measured E^C or E^M without applying any overpotential to the electrode and the potential was recorded until it became stable. The measured E^C and E^M values were recorded as this stabilized value. A temperature controlled mineral oil bath on a stirrer hot-plate was used to maintain a

constant temperature for the elevated temperature experiments. The electrochemical cell and Ag/AgCl reference electrode were placed in the oil bath 1 h prior to commencing any experiment. The potential of the reference electrode was repeatedly monitored by a secondary reference electrode (Hg/HgO at room temperature) while the experiment was underway. Tafel analyses were conducted under the same conditions with a scan rate of 1 mV s^{-1}. The potential window was from – 0.2 V to 0.2 V (vs the open circuit potential).

Predicted E^M and j^M

LSV tests for HMFOR and ORR were conducted following the method previously reported[4], with measurements performed at a scan rate of 10 mV s^{-1}. LSV curves presented in the main text and the supplementary file were subtracted by blank LSV curves, which were conducted at the same conditions without any reactants. For HMFOR, a $NaHCO_3$ and HMF solution was measured under an N_2 environment within the potential range of 0.2 V to 1.2 V (vs RHE). For ORR, a $NaHCO_3$ solution was measured with O_2 purging within the range of 0.9 V to 0.4 V (vs RHE). The elevated temperature set-up was identical to that described above.

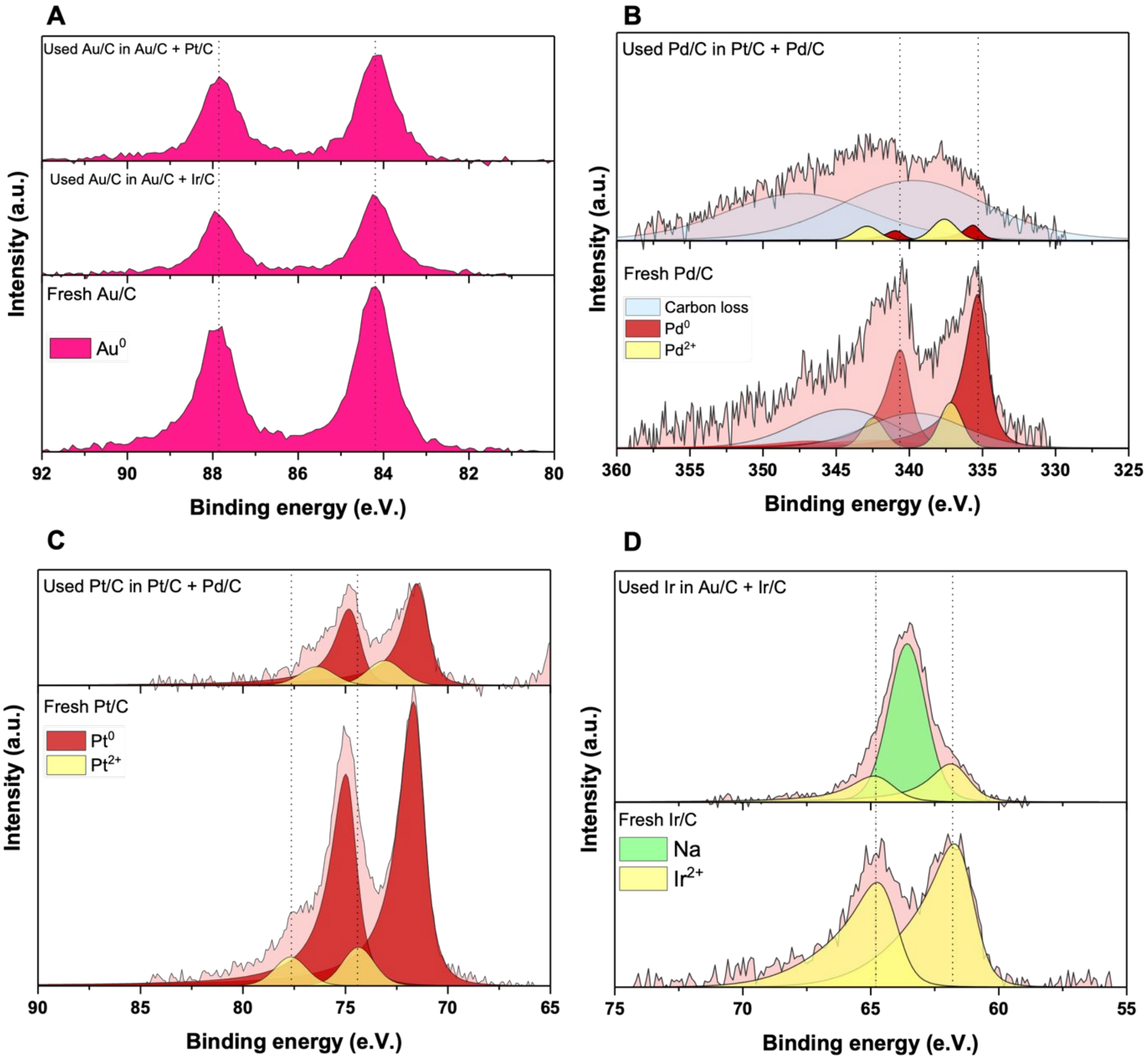
A
Used Au/C in Au/C + Pt/C
Used Au/C in Au/C + Ir/C
Fresh Au/C
Au0
Intensity (a.u.)
Binding energy (e.V.)
B
Used Pd/C in Pt/C + Pd/C
Fresh Pd/C
Carbon loss
Pd0
Pd2+
Intensity (a.u.)
Binding energy (e.V.)
C
Used Pt/C in Pt/C + Pd/C
Fresh Pt/C
Pt0
Pt2+
Intensity (a.u.)
Binding energy (e.V.)
D
Used Ir in Au/C + Ir/C
Fresh Ir/C
Na
Ir2+
Intensity (a.u.)
Binding energy (e.V.)

Figure S1. XPS spectra from fresh and used samples of catalysts. (**A**) Au 4*f* region from fresh monometallic Au/C, used Au/C + Ir/C physical mixture and used Au/C + Pt/C physical mixture, with the expected binding energy for the metallic Au highlighted by the dashed lines. (**B**) Pd 3*d* region for fresh monometallic Pd/C and used Pd/C + Pt/C physical mixture, with metallic Pd highlighted by the dashed lines. (**C**) Pt 4*f* region for fresh monometallic Pt/C and used Pt/C + Pd/C physical mixture, with Pt^{2+} peaks highlighted by the dashed lines. (**D**) Ir 4*f* region for fresh monometallic Ir/C and used Ir/C + Au/C physical mixture, with Ir^{2+} peaks highlighted by the dashed lines.

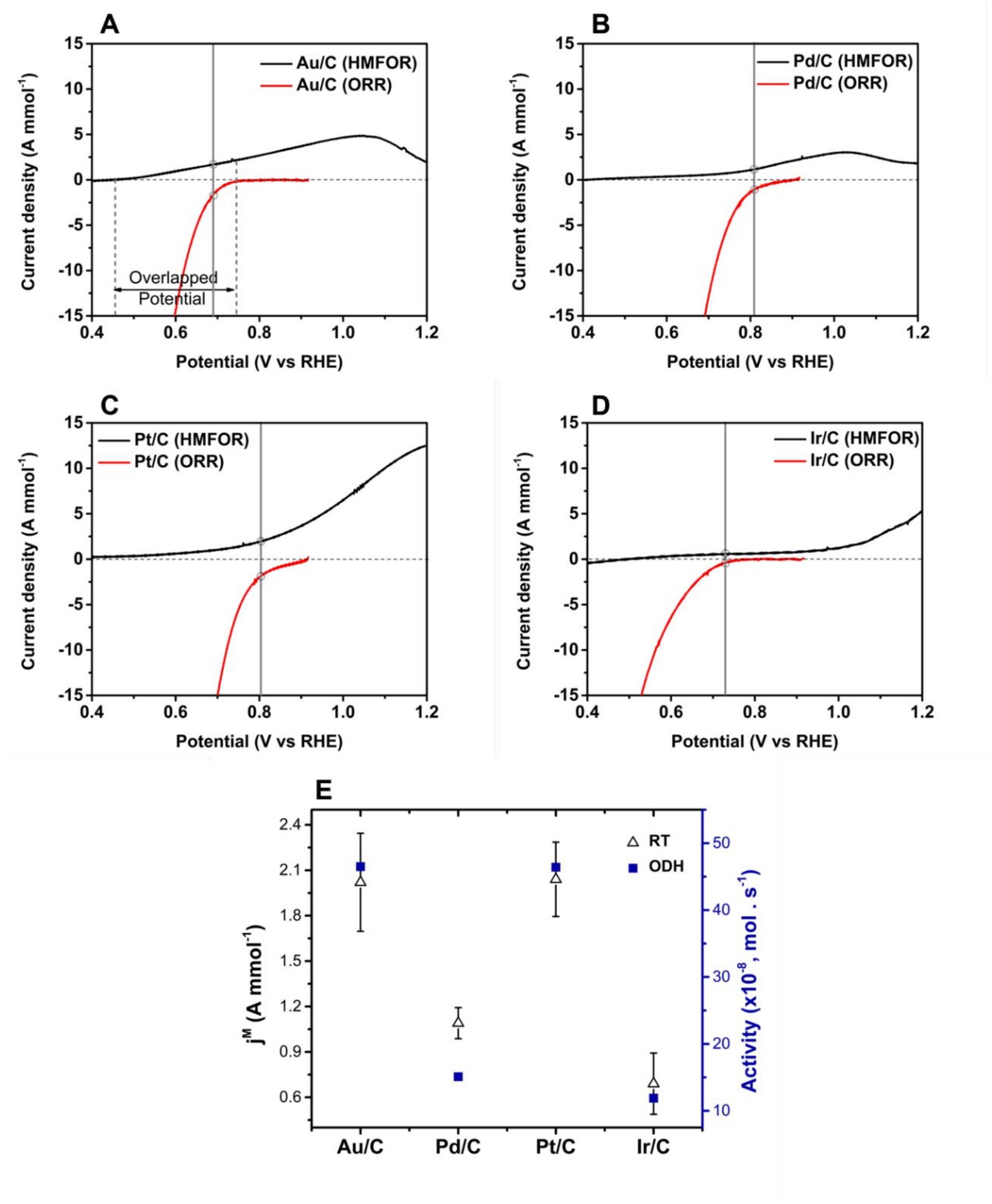

Figure S2. Linear sweep voltammetry curves of **(A)** Au/C, **(B)** Pd/C, **(C)** Pt/C, and **(D)** Ir/C for HMFOR (black line) and ORR (red line). Reaction conditions (HMFOR): 0.4 M $NaHCO_3$, 0.1 M HMF, 50 mL min^{-1} of N_2. Reaction conditions (ORR): 0.4 M $NaHCO_3$, 50 mL min^{-1} of O_2. All LSV curves were measured at room temperature with a scan rate of 10 mV s^{-1}. **(E)** Comparison between the j^M and ODH activity at room temperature.

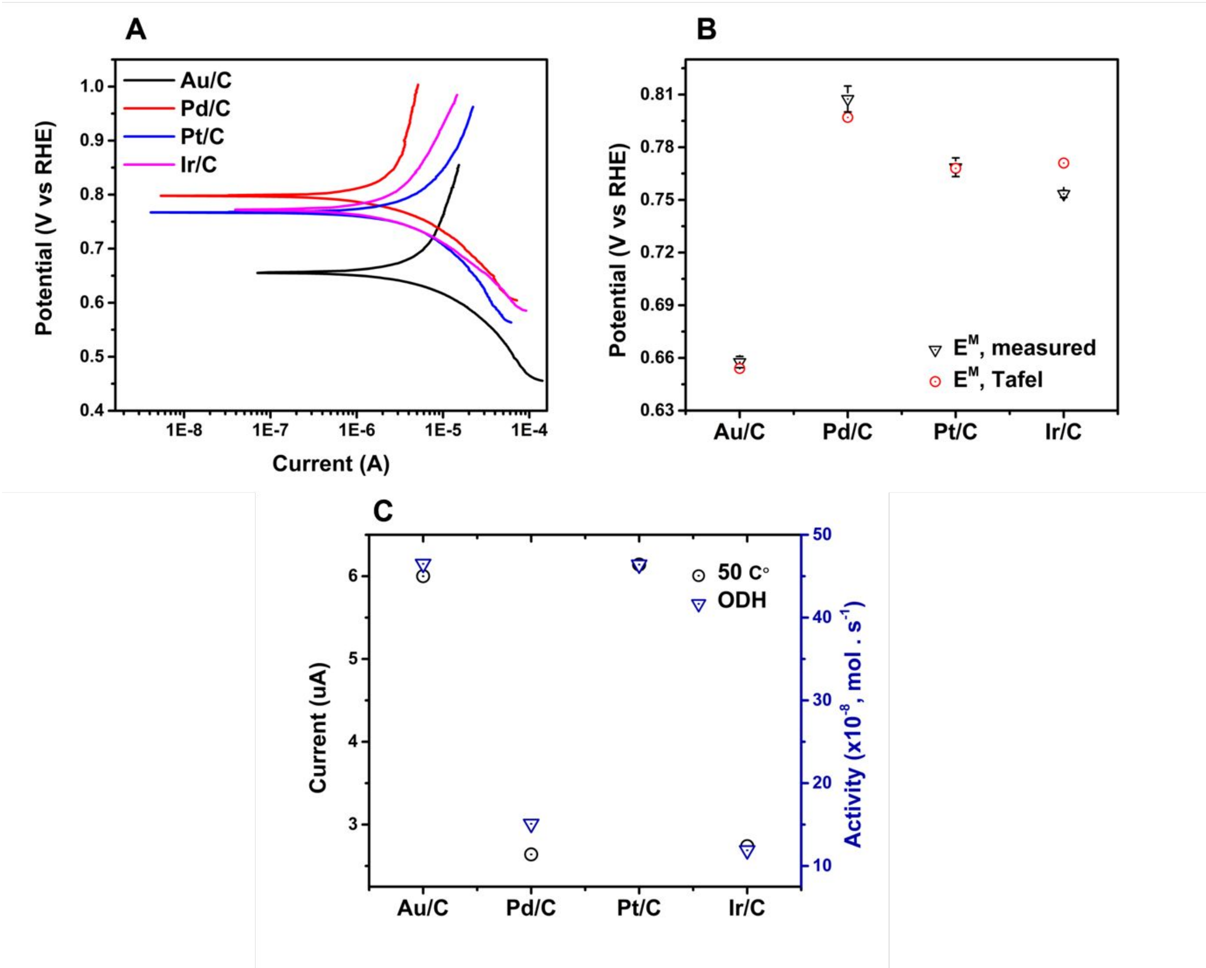

Figure S3. Tafel plots of (A) the various monometallic catalysts, (B) corresponding comparison of measured E^M and E^M values obtained from the Tafel plot, and (C) comparison of current and ODH activity. Reaction conditions: $NaHCO_3$ (0.4 M), HMF (0.1 M), O_2 (50 mL min^{-1}), 50 °C.

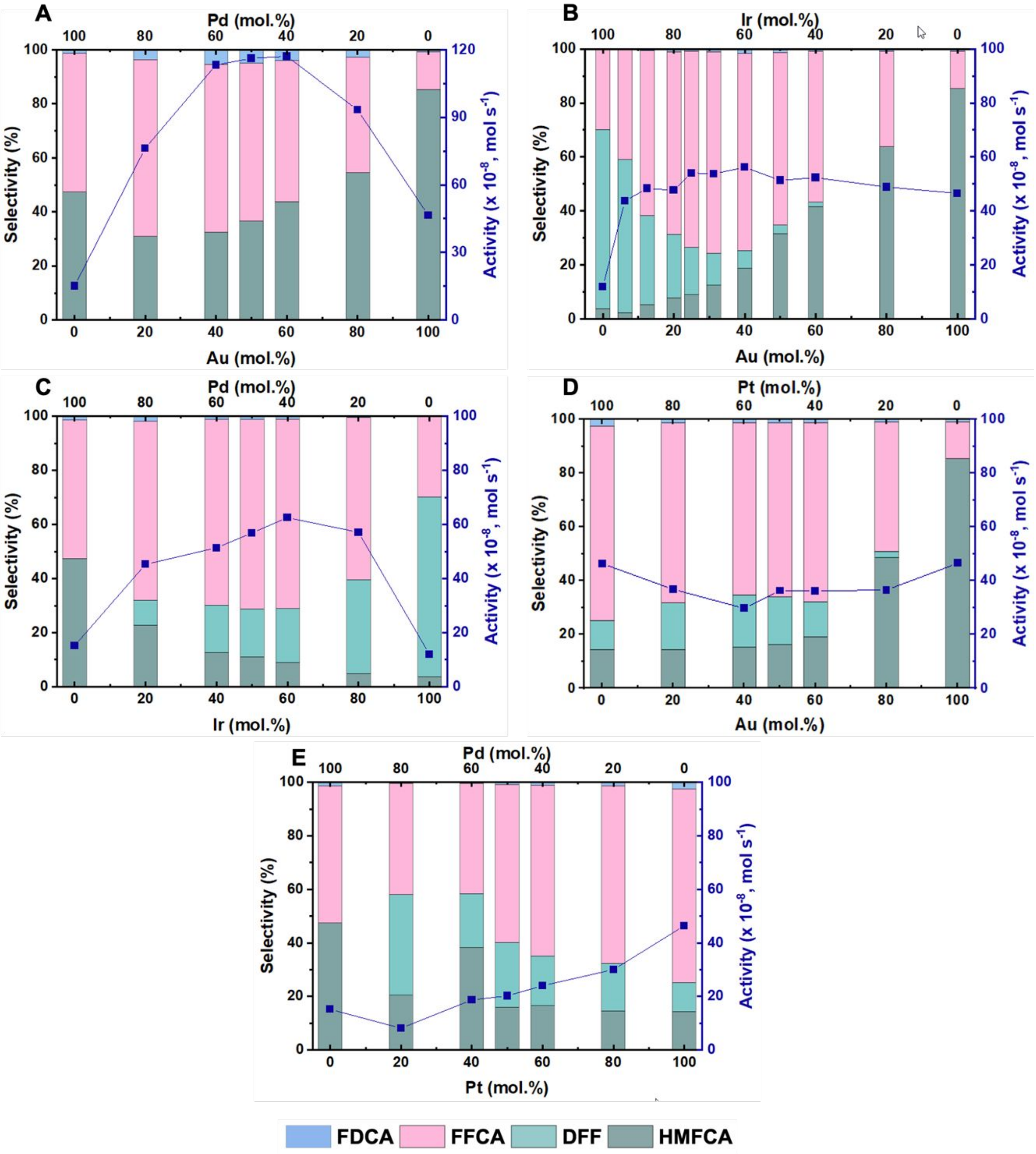

Figure S4. Activity and selectivity of the five physically mixed binary catalytic systems. **(A)** Au/C + Pd/C, reproduced with permission (*5*) **(B)** Au/C + Ir/C, **(C)** Pd/C + Ir/C, **(D)** Au/C + Pt/C, and **(E)** Pt/C + Pd/C. Reaction conditions: H_2O (16 mL), HMF (0.1 M), $NaHCO_3$ (0.4 M), 3 bar O_2, 80 ˚C, 30 minutes. The total metal content is constant in these bimetallic systems (HMF : metal = 200 : 1 (mol : mol)). The sum of the monometallic

activities are taken from the monometallic activities of each respective metal, for the specific molar quantity. All catalysts have approximately 1 wt.% total metal loading.

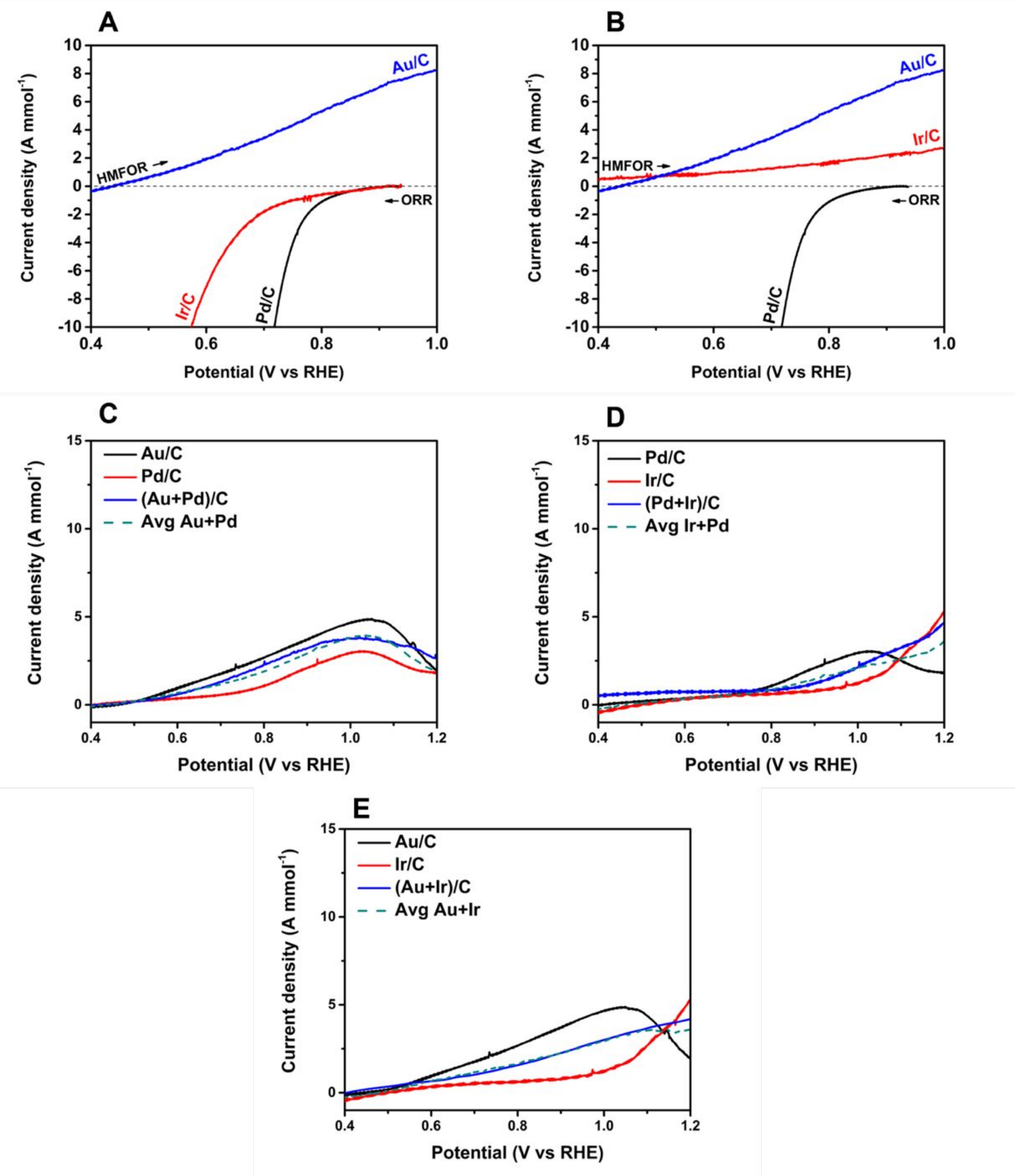

Figure S5. Linear sweep voltammetry curves at 50 ℃ of **(A)** Au/C (HMFOR), Pd/C (ORR), and Ir/C (ORR), and **(B)** Au/C (HMFOR), Ir/C (HMFOR), and Pd/C (ORR). **(A)** explains why ORR of Ir/C is limiting compared to that of Pd/C and **(B)** explains why HMF OR of Ir/C is limiting compared to that of Au/C. HMFOR LSVs at

room temperature (RT) for (**C**) Au/C, Pd/C, Au/C + Pd/C; **(D)** Pd/C, Ir/C, Ir/C + Pd/C; **(E)** Au/C, Ir/C and Au/C + Ir/C. The average value of the respective monometallic catalysts is also given (dashed lines**).** Reaction conditions (HMFOR): 0.4 M $NaHCO_3$, 0.1 M HMF, 50 mL min^{-1} of N_2. Reaction conditions (ORR): 0.4 M $NaHCO_3$, 50 mL min^{-1} of O_2.

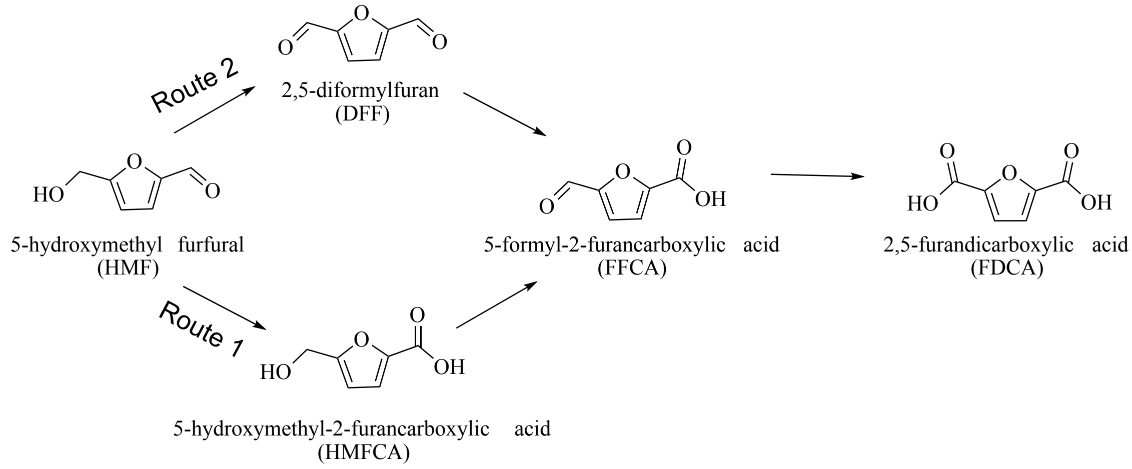

Scheme S1. The ODH of HMF can proceed via two pathways, with either the aldehyde (Route 1) or alcohol (Route 2) moiety transformed first to produce 2,5-diformylfuran (DFF) or 5-hydroxymethyl-2-furancarboxylic acid (HMFCA), respectively.

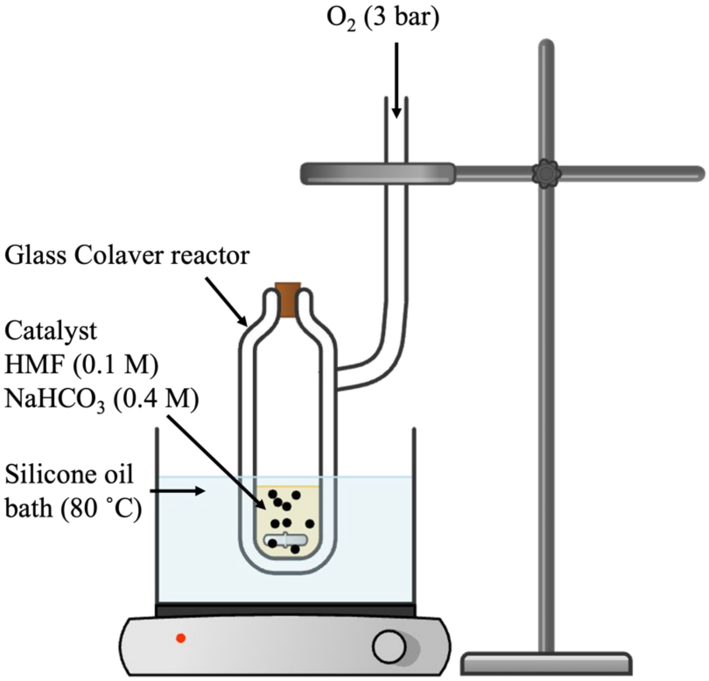

Scheme S2: Experimental configuration utilized for thermocatalytic testing. Reactions were conducted in a sealed glass Colaver reaction, with a constant feed of O_2.

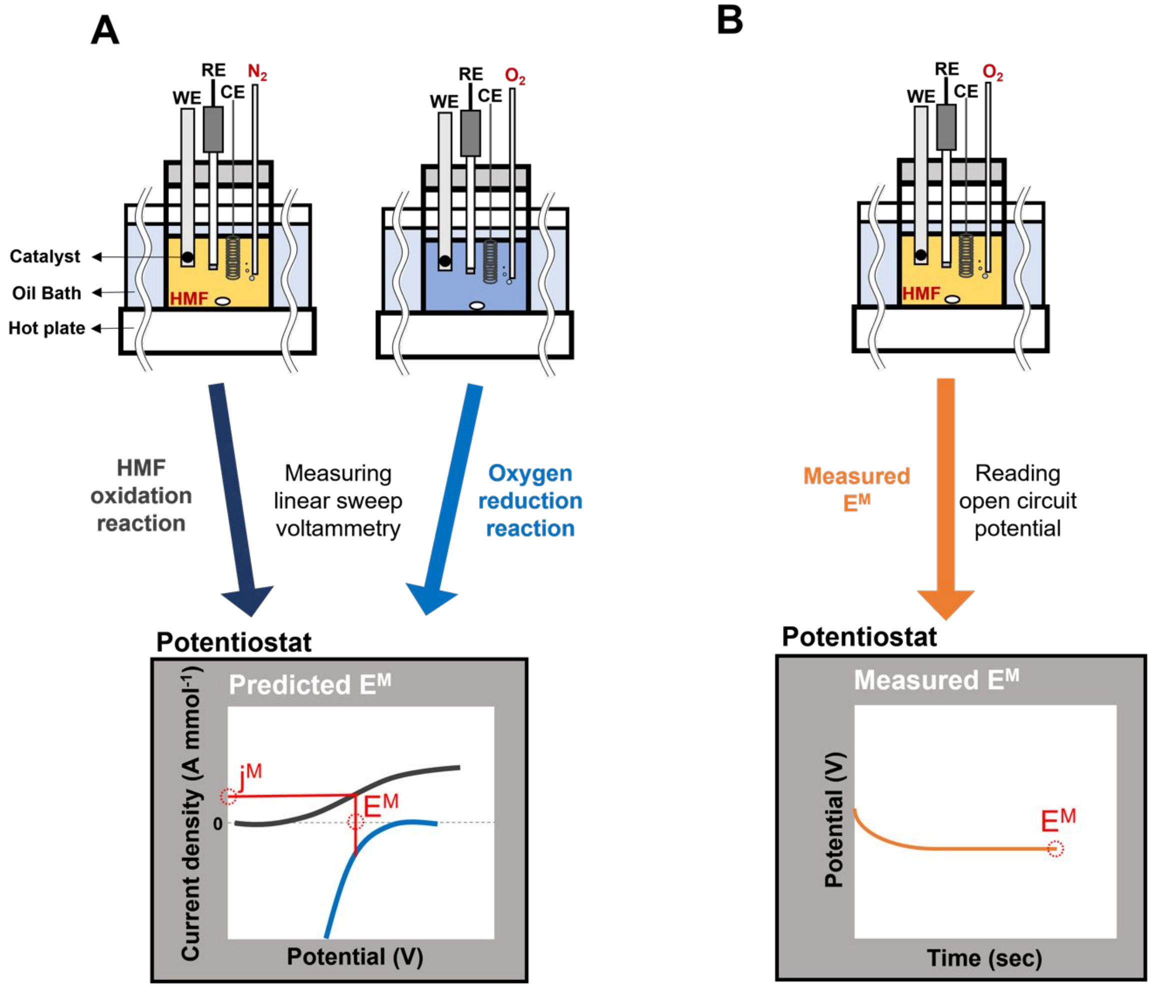

WE: working electrode (catalyst coated conductive glass)
RE: reference electrode (Hg/HgO or Ag/AgCl)
CE: counter electrode (Pt wire)

Scheme S3: Experimental configurations utilized in electrochemical measurements.

Table S1. ICP-MS analysis of each of the four monometallic catalysts used in this study. Target weight of 1 wt.%.

**Catalyst**	Au/C	Pd/C	Ir/C	Pt/C
**Weight percent of metal by ICP-MS**	1.01	1.04	0.94	1.01

Table S2. The ORR and HMFOR onset potential, and the predicted E^M and j^M values for each monometallic catalyst from Figure 4. The predicted E^M is calculated by taking the point at which the oxidation and reduction current are equal and normalizing this current by the mmol of metal present. Reaction conditions can be found in caption for Figure 4. Measured E^M values are included for comparison.

**Catalyst**	**ORR Onset Potential (V)**	**HMFOR Onset Potential (V)**	**Mixed Potential, E^M (V), measured***	**Mixed Potential, E^M (V), predicted****	**Mixed Current Density, j^M (A $mmol^{-1}$), predicted****
Au/C	0.72	0.47	0.658 ± 0.003	0.670 ± 0.013	3.1 ± 0.39
Pd/C	0.84	0.68	0.808 ± 0.007	0.800 ± 0.009	1.23 ± 0.28
Pt/C	0.86	0.56	0.769 ± 0.005	0.770 ± 0.014	3.1 ± 0.16
Ir/C	0.82	0.46	0.753 ± 0.002	0.730 ± 0.002	1.27 ± 0.09

*[1] The measured E^M was obtained with 0.1 M HMF, and 0.4 M $NaHCO_3$ solution under O_2.

**2 The predicted E^M and j^M values are taken from Figure 4 for each monometallic catalytic system.

Table S3. Maximum activity enhancement at the associated molar ratio observed for the three bimetallic catalytic systems that show evidence of a CORE effect. Full reaction data be found in Supplementary Figure. 6. Reaction conditions: H_2O (16 mL), HMF (0.1 M), $NaHCO_3$ (0.4 M), 3 bar O_2, 80 ˚C, 30 minutes.

**Catalytic Physical Mixture System (x & y)**	**Molar Ratio (x : y)**	**Activity Enhancement (x 10^{-8}, mol s^{-1})**
Au/C + Pd/C	1 : 1	90.9
Ir/C + Pd/C	3 : 2	51.8
Au/C + Ir/C	2 : 3	36.5

Supplementary References

1. M. Liu, R. Zhang, W. Chen, Graphene-supported nanoelectrocatalysts for fuel cells: Synthesis, properties, and applications. *Chem Rev.* **114**, 5117–5160 (2014).
2. Y. Shao, J. Liu, Y. Wang, Y. Lin, Novel catalyst support materials for PEM fuel cells: current status and future prospects. *J Mater Chem.* **19**, 46–59 (2008).
3. N. Fairley, V. Fernandez, M. Richard-Plouet, C. Guillot-Deudon, J. Walton, E. Smith, D. Flahaut, M. Greiner, M. Biesinger, S. Tougaard, D. Morgan, J. Baltrusaitis, Systematic and

collaborative approach to problem solving using X-ray photoelectron spectroscopy. *Appl Surf Sci Adv.* **5**, 100112 (2021).

4. S. Tanuma, C. J. Powell, D. R. Penn, Calculations of electron inelastic mean free paths. V. Data for 14 organic compounds over the 50–2000 eV range. *Surf Interface Anal.* **21**, 165–176 (1994).
